# Systems metabolic engineering, industrial biotechnology and microbial cell factories

**DOI:** 10.1186/1475-2859-11-156

**Published:** 2012-12-11

**Authors:** Sang Yup Lee, Diethard Mattanovich, Antonio Villaverde

**Affiliations:** 1 Metabolic and Biomolecular Engineering National Research Laboratory, Department of Chemical and Biomolecular Engineering (BK21 program), BioProcess Engineering Research Center, and Center for Systems and Synthetic Biotechnology, Institute for the BioCentury, KAIST, 291 Daehak-ro, Yuseong-gu, Daejeon, 305-701, Republic of Korea; 2 Department of Biotechnology, University of Natural Resources and Life Sciences (BOKU), Muthgasse 18, Vienna, 1190, Austria; 3 Austrian Centre of Industrial Biotechnology (ACIB), Vienna, Austria; 4 Institut de Biotecnologia i de Biomedicina, Universitat Autònoma de Barcelona, Bellaterra, Barcelona, 08193, Spain; 5 Department de Genètica i de Microbiologia, Universitat Autònoma de Barcelona, Bellaterra, Barcelona, 08193, Spain; 6 CIBER de Bioingeniería, Biomateriales y Nanomedicina (CIBER-BBN), Bellaterra, Barcelona, 08193, Spain

## 

Cell factories have been largely exploited for the controlled production of substances of interest for food, pharma and biotech industries. Although human-controlled microbial production and transformation are much older, the cell factory concept was fully established in the 80’s through the intensive public and private investment. Strongly empowered by the then nascent recombinant DNA technologies and supported by the approval of recombinant insulin [1,2], the principle of controlled biological production as a convenient source of difficult-to-obtain molecules (especially those of high added value) deeply penetrated the industrial tissue, soon becoming a widespread platform aiming at cost-effective large-scale production [3]. The first generation cell factories (mainly composed by plain strains of the bacterium *Escherichia coli* and the yeast *Saccharomyces cerevisae*) were soon replaced by engineered variants. Resulting from the application of untargeted mutagenesis and phenotypic selection, conventional genetic modification, metabolic engineering, and more recently by systems metabolic engineering that integrates metabolic engineering with systems biology and synthetic biology, new strains having much enhanced performance have been progressively developed [4-19]. In parallel, mammalian and insect cells for the production of high quality proteins, and other microbial species appealing from an industrial point of view due to their unusual physiological traits, have been incorporated to the cell factory family [20,21]. This comprises algae, fungi, psychrophilic bacteria and moss, among others [22-26]. On the other hand, a set of food-grade lactic acid bacteria are under development as emerging platforms in food microbiology but also as a novel source of metabolites and proteins [27-34]. The physiological diversity of the microbial world offers an intricacy of biosynthetic pathways from which novel bio-products, including nano- or micro-structured materials [35-37], offer promises in even more diverse applications.

Still, many substances and materials of industrial interest are nowadays produced by chemical synthesis, and the number of (recombinant) proteins approved for therapeutic use hardly reaches 200, a figure much lower than that initially presumed. However, both environmental concerns and medical and industrial needs strongly push towards a fully sustainable bio-production of a larger spectrum of substances. Then, how much limited is the economic feasibility of microbial production? Have the cell factories reached a plateau in their development? Is there more room for further exploitation of the microbial production?

Systems metabolic engineering [38,39] offers a set of methodological and strategic tools for the design and optimization of metabolic and gene regulatory networks for the efficient production of chemicals (from pharmaceuticals to bulk chemicals and fuels) and materials (from plastics to high value materials) [5-7,40]. Furthermore, creation of new metabolic pathways and fine tuning of the existing ones have become possible [8,15]. This is already allowing the production of substances so far reluctant to microbial production under industrial requirements and under cost-effective processes, allowing us to overcome limitations of microbial cell factories. *In silico* genome-scale metabolic modeling and simulation are playing increasingly important roles in system-wide identification of target genes and pathways to be manipulated to enhance production of desired products. Profiling of transcriptome, proteome, metabolome, and/or fluxome is also becoming a routine practice in metabolic engineering for the better understanding of cellular characteristics under given genetic and/or environmental perturbations. Through the system-wide optimized design and development of microbial cell factories, many industrially important chemicals and materials could be efficiently produced. Some example products include, but not limited to, alcohols other than ethanol including propanol, butanol and isobutanol, dicarboxylic acids such as succinic acid, fumaric acid, malic acid and adipic acid, diols such as 1,3-propanediol, 1,2-propanediol, 1,4-butanediol and 2,3-butanediol, diamines such as putrescine and cadaverine, and even polymers including polyhydroxyalkanoates, polylactic acid, spider silk protein, poly-gamma-glutamic acid, and many others [40]. It is expected that increasingly diverse chemicals and materials that are currently produced by petrochemical industry will be produced by employing microbial cell factories.

Improving cell factories by systems biotechnology to an industrially relevant level cannot be successfully reached by individual teams alone. A wide range of expertise is needed for the full development of a novel biotechnological process, including microbiology, chemistry, bioinformatics, biochemical engineering, but also agricultural and plant sciences and economics. After a proof of concept has shown the feasibility of a novel approach for cell factory engineering, large consortia or networks of fully skilled teams should take over in collaboration with Biotech companies to accomplish an economically feasible bioprocess. As an early example, the development of bacteria producing 1,3-propanediol from glucose has been well documented, and required the engineering of more than 70 genes, before process development and scale up were reasonably made [41]. Replacing materials which are produced from mineral oil by biobased products requires not only efficient technology but also the assessment of economical and ecological feasibility, as intensely studied for polylactic acid [42].

As the progress of industrial biotechnology did not accomplish the desired speed of development [43] academic researchers have teamed up with industry in publicly funded research centers worldwide. To name a few, the Joint BioEnergy Institute (JBEI) in the San Francisco Bay Area, the Novo Nordisk Foundation Center for Biosustainability (CFB) at the Technical University of Denmark, the Metabolic and Biomolecular Engineering Laboratory (MBEL) of the Korea Advanced Institute of Science and Technology (KAIST), the Dutch Klyuver Centre for Genomics of Industrial Fermentation, or the Austrian Centre of Industrial Biotechnology (ACIB) share the mission to speed up development of industrial bioprocesses by joining forces of different academic disciplines with industrial collaborators.
